# Mavoglurant (AFQ056) for the treatment of levodopa-induced dyskinesia in patients with Parkinson’s disease: a meta-analysis

**DOI:** 10.1007/s10072-021-05319-7

**Published:** 2021-05-20

**Authors:** Ahmed Negida, Hazem S. Ghaith, Salma Yousry Fala, Hussien Ahmed, Eshak I Bahbah, Mahmoud Ahmed Ebada, Mohamed Abd Elalem Aziz

**Affiliations:** 1 Medical Research Group of Egypt, Cairo, Egypt; 2 Faculty of Medicine, School of Medicine, Zagazig University, Sharkia, Zagazig, 44523 Egypt; 3 School of Pharmacy and Biomedical Sciences, University of Portsmouth, Portsmouth, UK; 4 Faculty of Medicine, Al-Azhar University, Cairo, Egypt; 5 Faculty of Medicine, Suez Canal University, Ismailia, Egypt; 6 Faculty of Medicine, Al-Azhar University of Damietta, Damietta, Egypt; 7 Neuropsychiatry Department, Omr Shahin Mental Hospital, Cairo, Egypt

**Keywords:** Parkinson's disease, Mavoglurant, Levodopa

## Abstract

**Background:**

Mavoglurant (AFQ056), a selective metabotropic glutamate receptor 5 (mGluR5) inhibitor, was tested for t levodopa-induced dyskinesia (LID) in patients with Parkinson’s Disease (PD). However, clinical trials showed inconsistent results regarding the efficacy of mavoglurant in treating LID in patients with Parkinson's disease (PD).

**Methods:**

A computer literature search of PubMed, Scopus, Web of science, and Cochrane CENTRAL was conducted until March 2021. We selected relevant randomized controlled trials comparing mavoglurant to placebo. Study data were extracted and pooled as mean difference (MD) in the meta-analysis model.

**Results:**

Six RCTs were included in this meta-analysis with a total of 485 patients. Mavoglurant was not significantly superior to placebo in terms of the “off-time” (MD −0.27 h, 95% CI −0.65 to 0.11), “on time” (MD 0.29 h, 95% CI −0.09 to 0.66), Lang-Fahn activities of daily living dyskinesia scale (MD −0.95, 95% CI −1.98 to 0.07), UPDRS-III (MD −0.51, 95% CI −1.66 to 0.65), or UPDRS-IV (MD −0.41, 95% CI −0.85 to 0.03). However, the pooled modified abnormal involuntary movement scale favored the mavoglurant group than the placebo group (MD −2.53, 95% CI −4.23 to −0.82).

**Conclusions:**

This meta-analysis provides level one evidence that mavoglurant is not effective in treating the LID in patients with PD.

**Supplementary Information:**

The online version contains supplementary material available at 10.1007/s10072-021-05319-7.

## Introduction

Parkinson's disease is a common neurological disorder affecting about 1–3% above 60 years population [1]. The loss of dopaminergic neurons and the formation of Lewy bodies are the cardinal pathological features of PD. PD symptoms include rigidity, tremors, and dyskinesia due to decreased dopamine levels in the basal ganglia of PD patients. Levodopa, a precursor of dopamine, is considered as the standard of care for patients with early PD. However, PD patients do not respond optimally to levodopa. The wearing off of the drug and motor fluctuations influence the quality of life of PD patients.

At 5 years after initiation of levodopa treatment, about 50% of PD patients suffer from levodopa-induced dyskinesia (LID), requiring dose adjustments to balance the motor benefit against the related complications [2, 3]. Amantadine, a NMDA blocker, is an effective drug to treat LID; however, its short-term efficacy warranted the development of alternative add-on agents with long-term durability [4, 5]. There is an unmet clinical need for adjuvant treatments to treat LID and allow reductions in levodopa dose with long term durability and without influencing the motor benefit of the levodopa treatment.

The mechanism of LID is not yet elucidated; however, the literature suggests that the glutamatergic pathway has a role in the emergence of LID [2, 6]. Increased glutamatergic signaling was found in the striatum of basal ganglia [7]. Additionally, the modulation of glutamatergic signaling by mGluR5 antagonists was found to be effective in reducing the LID in animal models [8]. Mavoglurant (AFQ056), a selective mGluR5 inhibitor, was developed to treat LID by decreasing the glutamatergic signaling in the striatum [9].

Multiple clinical trials were conducted to assess the safety and efficacy of mavoglurant for the treatment of LID in PD patients. Berg *et al*. [10] found a significant improvement in the Modified abnormal involuntary movement scale (mAIMS) and unified Parkinson's disease rating scale (UPDRS)-IV scores in the mavoglurant group compared to the placebo group. However, a recent study by Trenkwalder *et al*. [11] showed no evidence of benefit from mavoglurant.

There is a lack of class-one evidence about the safety and efficacy of mavoglurant for the treatment of LID in patients with PD. Hereby, we evaluated the safety and efficacy of mavoglurant compared to placebo for the treatment of LID in PD patients using data from randomized controlled trials pooled in the context of meta-analysis.

## Methods

We followed the Preferred Reporting Items of Systematic Reviews and Meta-analysis during the preparation of this manuscript [12]. The methods and analyses were conducted in strict accordance with the guidelines of the Cochrane Handbook of Systematic Review and Meta-analysis and the Methods Guide for Comparative Effectiveness Reviews [13, 14].

### Eligibility criteria

Studies that fit all of the following criteria were included in the meta-analysis:

1. Population: Studies whose population was patients with idiopathic PD (diagnosed using the UK PD society Brain Bank Criteria) [12] and suffering from moderate to severe LID (defined as those whose Unified Parkinson’s Disease Rating Scale-IV item 32 >1 and item 33 >2).
2. Intervention: Studies where patients receive AFQ056 as experimental drug (all doses are considered).
3. Comparator: Studies where the control group received placebo
4. Study design: Studies that were described as prospective randomized controlled trials.

We excluded studies on drugs other than AFQ056.

### Literature search

We searched electronic databases: PubMed, Scopus, Web of science, and Cochrane CENTRAL through March 2021, using the following keywords: “(AFQ056 OR mavoglurant OR mGluR5) AND Parkinson’s disease”. No limitations or filters were employed.

### Study selection

Results of databases search were screened by titles and abstracts; then full-text articles of eligible abstracts were retrieved for further eligibility screening and assessment of the reliability of data for analysis.

### Data collection process and data items

An online data extraction sheet was constructed. The data extraction includes the following domains: (1) study ID, (2) study year, (3) country, (4) study design, (5) follow up duration, (6) drug dose, (7) population definition, (8) inclusion and exclusion criteria, (9) sample size, (10) baseline characteristics, (11) available data of outcome measures (pre, post, and change from baseline), and (12) quality assessment domains.

### Risk of bias in individual studies

The Cochrane risk of bias assessment tool [13] is a recommended checklist for assessing the risk of bias in RCTs. In this tool, each RCT is assessed for the possibility of the following risks: (1) selection bias, by assessing the methods of random sequence generation and the concealment of patient allocation; (2) performance bias, by assessing the blinding of participants and study personnel; (3) detection bias, by assessing the blinding methods of outcome assessment process; (4) attrition bias, by evaluating the magnitude and impact of incomplete outcome data and whether incomplete data were handled by appropriate statistical analysis techniques; (5) reporting bias, by assessing the selectivity of reporting study outcomes based on the prespecified methods in the clinical trial registration; and (6) any other source of bias that might have influenced the study data.

### Efficacy measures

The efficacy of drugs treating LID is assessed for the following outcomes.

#### Patient-reported home diaries which give the following outcomes

Patient-reported diaries include information about the duration of “off-time”, “on time”, “on time without dyskinesia”, “on time with nontroublesome dyskinesia”, and “on time with troublesome dyskinesia”.

#### Modified abnormal involuntary movement scale (mAIMS)

MAIMS [14] is a composite score of dyskinesia rating in seven body parts.

#### UPDRS part 3 and part 4 (items 32 and 33)

The unified Parkinson’s disease rating scale [15] is a reliable score of four parts to assess the severity of PD symptoms. Part 3 indicates the motor score, whereas part 4 indicates the severity of PD complications, with items 32 and 33 referring to the duration and severity of dyskinesia, respectively.

#### Lang-Fahn activities of daily living dyskinesia scale (LFADLDS)

Lang-Fahn activities of daily living dyskinesia scale focus on patient perceptions of disability related to dyskinesia. An ordinal scale (0, 0 representing no dyskinesia and 4 representing inability to perform the task) assesses. The patient’s report on five activities of daily living potentially impacted by dyskinesia at their maximum severity over the past few days (handwriting or drawing, cutting food and handling utensils, dressing, hygiene, and walking).

### Synthesis of results

Because all efficacy outcomes are reported as continuous data, for each efficacy measure, the mean difference (MD) between the two groups from the baseline to endpoint, with its standard error (SE) were pooled in the DerSimonian-Laird random effect model. In case of studies reporting data in multiple time points, we considered the last endpoint. The overall MD was interpreted with the consideration that efficacy measures are in different directions; the improvement in mAIMS, UPDRS IV, UPDRS III, “off time”, and “on time with troublesome dyskinesia” is indicated by decreased MD while the improvement in “on time without dyskinesia” and “on time with nontroublesome dyskinesia” is indicated by increased MD. For the safety analysis, we analyzed adverse events reported with an incidence ≥5% in the included studies. The proportion of adverse events to the total number of patients in each group were pooled as relative risk between the two groups in the DerSimonian-Laird random effect model. Heterogeneity (noncombinability) of studies and subgroups was examined by visual inspection of the forest plot and assessed by the Cochrane Q and I^2^ tests using RevMan version 5.3 for windows.

### Calculation of missing data

When the mean difference from baseline to endpoint was not provided, we calculated it from the pretreatment and posttreatment means [MD = Posttreatment − pretreatment]. Then, we calculated mean difference between the AFQ056 and placebo groups as follows: [MD = MDexperiemental − MDplacebo]. When the SE of mean difference was not provided, we calculated it from the standard deviation [SE = SD/ √ *n*], 95% confidence interval [(upperlimit − lowerlimit)/3.92], or 90% CI [(upperlimit − lowerlimit)/3.29]. For studies and groups with sample size less than 60 patients, the numbers (3.92 and 3.29) were substituted by a value from the table of *t* distributions with degrees of freedom equal to the group sample size minus 1.

### Risk of bias across studies

In order to explore the publication bias across studies, we constructed funnel plots to present the relationship between effect size and precision. Evidence of publication bias was assessed by the following: (1) Egger’s regression test and (2) the Begg and Mazumdar rank correlation test (Kendall’s tau).

## Results

### Study selection

The literature search of PubMed, Scopus, Web of science, and Cochrane CENTRAL yielded 15 records. Following titles and abstract screening, four articles describing six randomized controlled trials with a total of 485 patients were included in the meta-analysis [10, 11, 15, 16]. The flow of the study selection process is shown in the PRISMA flow diagram in Supplementary File 1.

### Study characteristics

The duration of follow-up in the studies ranged from 2 weeks in the Berg *et al* (2010) [7](study 1 and study 2) to 13 weeks in the study by Stocchi *et al*(2013)[17]. Patients in studies by Berg *et al* (2010) [7]and Kumar *et al* (2016) [16]received daily doses of mavoglurant ranging from 20 to 300 mg, while patients in the study by Stocchi *et al* (2013) were classified into five subgroups receiving mavoglurant doses of 25, 50, 100, 150, or 200 mg. In all studies, PD patients remained on the levodopa treatment during the study period.

The population of these studies was homogenous. All studies enrolled patients with Parkinson’s disease (diagnosed according to UK PD society brain bank criteria) with moderate to severe LID as indicated by the UPDRS-IV (items 32 and 33). All studies excluded patients with (1) history of psychological problems, (2) prior surgical treatment for PD, and (3) use of antipsychotic or antidyskinetic drugs 15 days prior to randomization. Summary and baseline characteristics of populations of these studies are shown in Table 1. The quality of included studies was acceptable according to the Cochrane Risk of Bias assessment tool.

**Table 1:** Baseline characteristics of the study population in the included studies

Study ID	Design	Final endpoint	Group	N	Age*	Male	mAIMS*	UPDRS-III*	UPDRS-IV (items 32 and 33)*
Berg 2010 (Study 1)	RCT	2 weeks	Mavoglurant	15	60.7 (10.5)	60%	11.4 (4.79)	26.6 (14.78)	4.7 (1.03)
			Placebo	16	61.4 (10.2)	43.8%	9.1 (3.87)	22.1 (9.77)	4.1 (0.89)
Berg 2010 (Study 2)	RCT	3 weeks	Mavoglurant	14	65.6 (7.5)	64.3%	16 (4.07)	26.1 (16.67)	5.4 (0.95)
			Placebo	14	66.1 (6.5)	57.1%	16.3 (4.32)	26 (9.68)	4.8 (0.89)
Stocchi 2013	RCT	13 weeks	Mavoglurant 20 mg	22	66.2 (8.16)	45.5%	14.5 (4.64)	16.6 (8.13)	5.1 (0.89)
			Mavoglurant 50 mg	22	66.4 (7.96)	59.1%	12.9 (5.03)	18.6 (7.97)	4.9 (0.01)
			Mavoglurant 100 mg	23	65.6 (9.47)	60.9%	13.3 (5.25)	18.5 (9.65)	4.8 (0.8)
			Mavoglurant 150 mg	22	66 (10.54)	63.6%	13.9 (5.08)	18.9 (9.25)	4.8 (0.73)
			Mavoglurant 200 mg	44	63.4 (8.98)	54.5%	13.4 (4.91)	17.5 (9.31)	5 (0.88)
			Placebo	64	64.8 (8.17)	46.9%	13.5 (4.68)	16.9 (9.39)	4.9 (0.82)
Kumar 2016	RCT	5 weeks	Mavoglurant	7	61.3 (8.98)	57.1%	NR	22.3 (15.32)	NR
			Placebo	7	61.4 (6)	71.4%	NR	16.1 (7.8)	NR
Trenkwalder 2016 (study 1)	RCT	12 weeks	Mavoglurant 100 mg	36	65.9 (6.97)	52.8%	12.5 (4.88)	20.5 (10.13)	4.8 (0.87)
			Placebo	25	66.6 (7.04)	60.0%	12.8 (5.08)	19.5 (7.91)	4.8 (0.66)
Trenkwalder 2016 (study 2)	RCT	12 weeks	Mavoglurant 150 mg	39	64.4 (8.68)	56.4%	12.38 (4.178)	18.05 (9.409)	4.67 (0.898)
			Mavoglurant 200 mg	78	64.4 (8.84)	52.6%	12.59 (5.062)	20.26 (11.319)	4.82 (0.833)
			Placebo	37	64.2 (9.02)	56.8%	11.73 (5.064)	19.32 (9.548)	4.81 (0.811)

*Continuous outcomes presented as mean (SD). *mAIMS*, modified abnormal involuntary movement scale; *UPDRS*, unified Parkinson’s disease rating scale; *RCT*, randomized controlled trials; *NR*, not reported

### Drug efficacy

#### Off-time

**Fig. 1:**
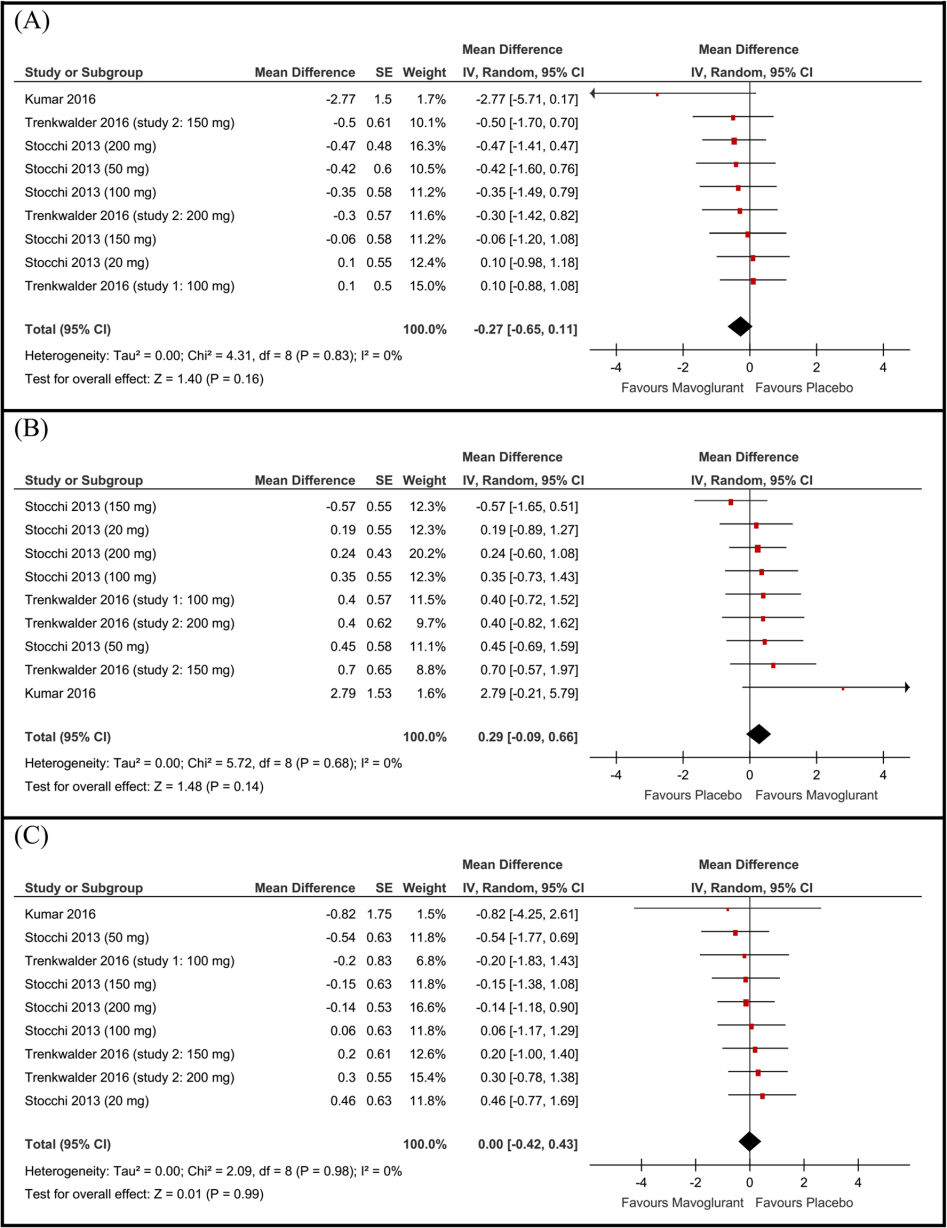
Forest plot of the mean difference and 95% confidence intervals of the **A** off time, **B** total on time, and **C** on time with troublesome dyskinesia

The overall mean difference between the two groups from baseline to endpoint in terms of change in "Off time" did not favor either of the two groups (MD −0.27 h, 95% CI [−0.65 to 0.11], Fig. 1A). Pooled studies were homogenous (*P*=0.83).

#### Total on time

The pooled mean difference of change in the total “on-time” did not favor either of the two groups (MD 0.29 h, 95% CI [−0.09 to 0.66], Fig. 1B). Pooled studies were homogenous (*P* = 0.68).

#### On time without dyskinesia

The study by Kumar *et al*.(2016) [15]was the only on that reported the “on time without dyskinesia”. In this study, patients in the mavoglurant group had more improvement in the “on-time without dyskinesia” compared with the placebo group (mean change from baseline to endpoint in both groups: 3.83 vs. 1.42 h, respectively).

### On time with troublesome dyskinesia

None of the included studies reported a statistically significant improvement in the “on time with troublesome dyskinesia” with the mavoglurant compared to placebo. The pooled mean difference of change in the “on time with troublesome dyskinesia” did not favor either of the two groups (MD 0.00 h, 95% CI [−0.42 to 0.43], Fig. 1C). Pooled studies were homogenous (*P* = 0.98).

### mAIMS

**Fig. 2:**
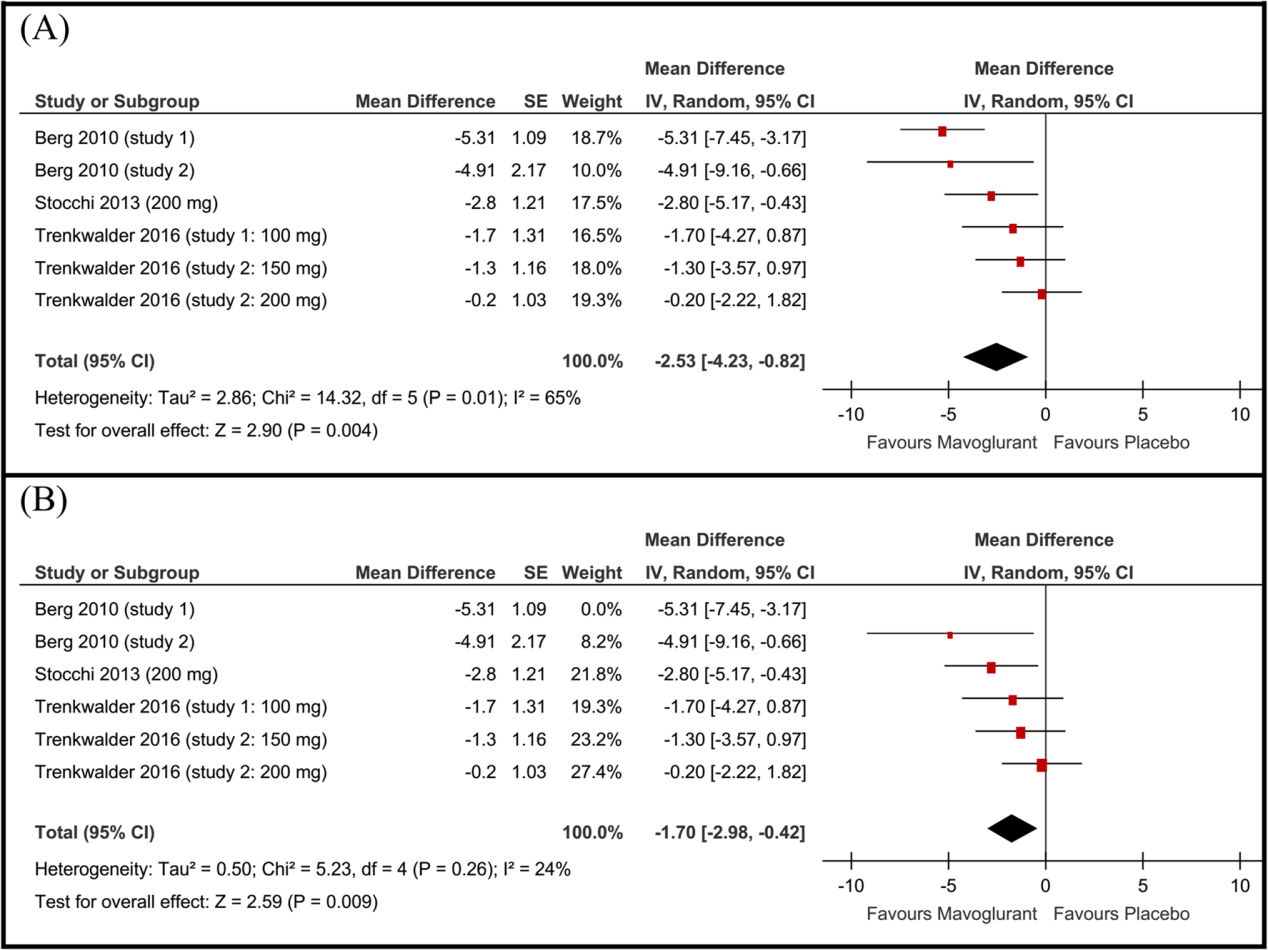
Forest plot of the mean difference and 95% confidence intervals of the **A** modified abnormal involuntary movement scale (mAIMS) score and **B** mAIMS score after sensitivity analysis excluding Berg study 1

In the mavoglurant group of studies 1 and 2 by Berg et al (2010) [7], and Stocchi *et al* (2013) [17] (group of 200 mg), the improvement in mAIMS score was significantly higher than that in the placebo groups. For the subgroups in the other studies, the improvement in mAIMS within the mavoglurant group was higher but not statistically significant. When combined in the meta-analysis model, the pooled mean difference of change in the mAIMS favored the mavoglurant group over the placebo group (MD −2.53, 95% CI [−4.23 to −0.82], Fig. 2A). Pooled studies were not homogenous (*P*=0.01, I-square=65%). Therefore, we performed a sensitivity analysis in seven scenarios excluding one study/subgroup in each scenario. Heterogeneity was best resolved by excluding study 1 by Berg *et al* (2010) [7] (*P*=0.26, I^2^ =24%). Following resolving heterogeneity, the effect estimate remained in favor of the mavoglurant group over the placebo group (MD −1.70 point, 95% CI [−2.98 to −0.42], Fig. 2B).

### Lang-Fahn activities of daily living dyskinesia scale (LFADLDS)

**Fig. 3:**
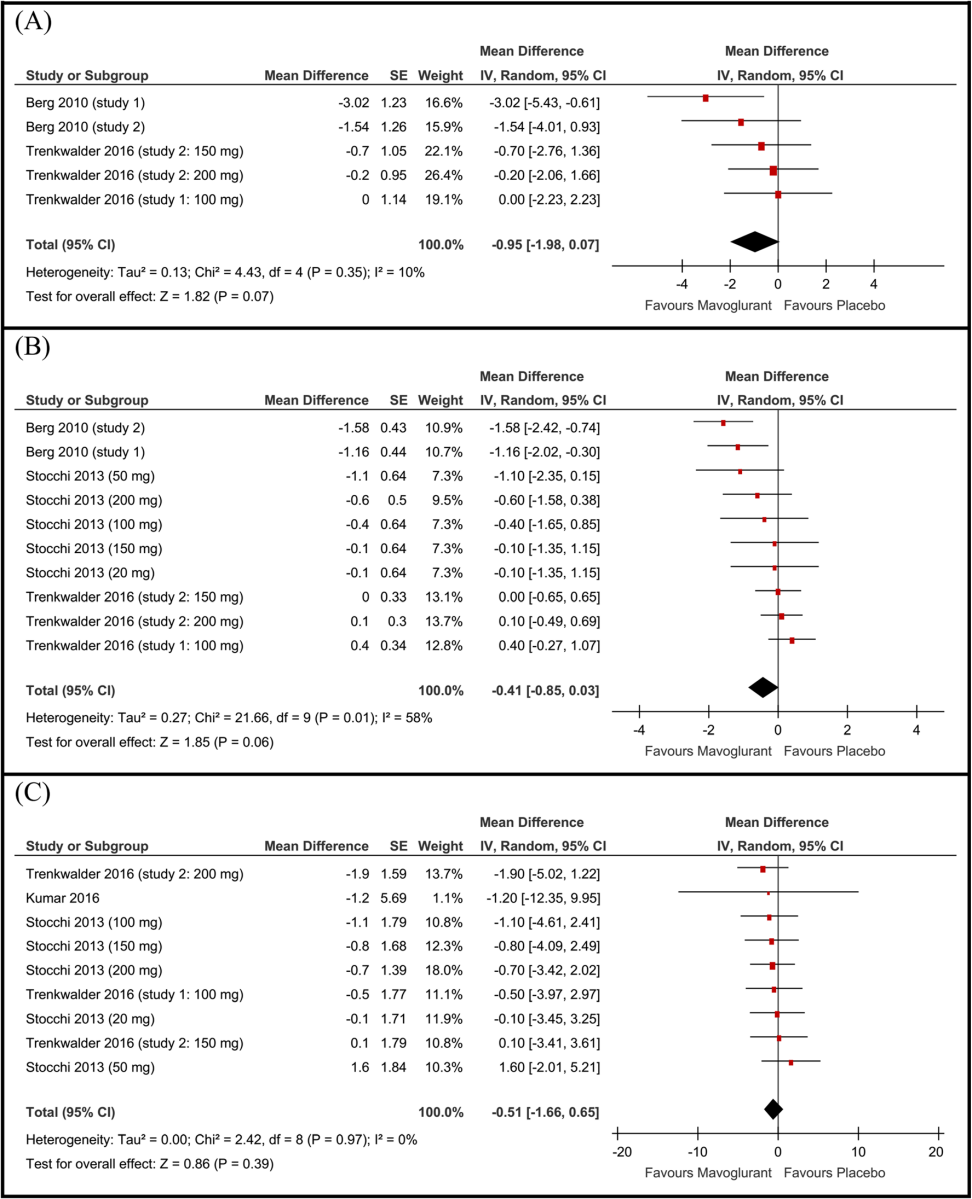
Forest plot of the mean difference and 95% confidence intervals of the **A** Lang-Fahn activities of daily living dyskinesia scale, **B** UPDRS-IV items 32 and 33, and **C** UPDRS-III motor score

The overall mean difference of change in LFADLDS from baseline to end point did not favor either of the two groups (MD −0.95, 95% CI [−1.98 to 0.07], Fig. 3A), a *P* value of 0.07 illustrated a trend toward favoring the mavoglurant group. Pooled studies were homogenous (*P*=0.35).

### UPDRS-IV (items 32 and 33)

Compared to the placebo group, the mavoglurant group achieved better scores on the UPDRS-IV (items 32 and 33). This difference was statistically significant in the Berg *et al*. (2010) [7] (study 1 and study 2). However, the pooled mean difference of change in the UPDRS-IV (items 32 and 33) did not favor either of the two groups (MD −0.41 point, 95% CI [−0.85 to 0.03], Fig. 3B).

### UPDRS-III

All studies showed no statistically significant difference between the mavoglurant and placebo groups in terms of the change in UPDRS-III. The pooled mean difference of change in the UPDRS-III did not favor either of the two groups (MD −0.51 point, 95% CI [−1.66 to 0.65], Fig. 3C). Pooled studies were homogenous (*P*=0.97).

### Adverse events

Of the seven analyzed adverse events, only dizziness showed a statistically significant risk in the mavoglurant group compared to the placebo group (RR 4.19, 95% CI [1.83 to 9.64]). In all studies, patients in the mavoglurant group experienced more hallucinations, euphoria, and nausea than patients in the placebo group, however, the pooled RR was not statistically significant. Effect estimates of the adverse events with their 95% confidence intervals are shown in Table 2.

**Table 2:** Summary of the pooled risk ratios (RR) between the mavoglurant group and placebo group in all reported adverse events

Adverse event	N of RCTs	N patients	RR [95% CI]
Dizziness	5	471	4.19 [1.83 to 9.64]
Dyskinesia	6	585	0.74 [0.46 to 1.17]
Euphoria	3	73	3.70 [0.64 to 21.29]
Fatigue	5	471	2.11 [0.72 to 6.15]
Insomnia	4	426	1.90 [0.58 to 6.23]
Hallucination	2	211	4.55 [0.86 to 24.04]
Nausea	4	274	2.02 [0.48 to 8.46]

*RCT*, randomized controlled trial

### Risk of bias across studies

Our meta-analysis included fewer than 10 studies. Therefore, the assessment of publication bias using the funnel plot method and Egger’s test will not be reliable as stated by Egger’s and colleagues.

## Discussion

### Summary of evidence

The present meta-analysis provides class one evidence that mavoglurant as an add-on treatment does not improve LID as reported by patient diaries and UPDRS-IV (items 32 and 33). However, it was associated with a significant improvement in the dyskinesia scores as measured by the mAIMS score. Additionally, mavoglurant did not influence the antiparkinsonian effects of levodopa as measured by the UPDRS-III. In terms of safety, the drug was associated with a fourfold increase in the incidence of dizziness compared to the placebo group.

### Previous studies

The effect estimates produced in our meta-analysis are consistent with the previous randomized controlled trials in terms of LID measured by patient diaries. However, in terms of mAIMS and UPDRS-IV (items 32 and 33), results of randomized controlled trials were controversial. Berg *et al*.(2010) [7] described two RCTs (studies 1 and 2) on PD patients with moderate to severe and severe LID, respectively. Unlike other studies, patients in the studies by Berg *et al*.(2010) [10] showed statistically significant improvements in mAIMS and UPDRS-IV (items 32 and 33). This could be explained by the patients in the studies by Berg *et al*.(2010) [10] having poorer baseline UPDRS-III scores than those in other studies (baseline characteristics are shown in Table 1). This observation highlights that mavoglurant might be effective for patients with UPDRS-III >22. In the study by Stocchi *et al*.(2013) [16], patients receiving 200 mg mavoglurant showed statistically significant improvements in mAIMS scores compared to those receiving placebo; however, this improvement was not significant for the other doses (20, 50, 100, and 150 mg). The pooled analysis showed a small but statistically significant improvement in mAIMS score, but no significant improvement was found in the UPDRS-IV (items 32 and 33).

### Strengths of the study

Our meta-analysis has multiple strengths: (1) the literature search strategy was rigorous; (2) the research question was supported by clear eligibility criteria; (3) each step in the review was done by multiple reviewers to ensure accuracy; (4) we followed the Preferred Reporting Items of Systematic Review and Meta-analysis during the preparation of this manuscript; (5) we conducted this meta-analysis in strict accordance with the guidelines of Cochrane Handbook of systematic reviews and meta-analysis; and (6) the present data were generated from randomized controlled trials with high internal validity and acceptable quality as indicated by the risk of bias assessment.

### Limitations of the study

The limitations of this meta-analysis are the following: (1) the limited number of studies did not allow for investigation of the effect of dose on patient outcomes and (2) the limited sample size in the present RCTs.

### Implications for future research

Based on this meta-analysis, we recommend no future randomized controlled to investigate the efficacy mavoglurant for PD patients suffering from LID. We do not recommend the use of this drug in the clinical practice.

### Conclusions

Current evidence does not support the efficacy of mavoglurant for the treatment of LID in PD patients. We do not recommend the use of this drug for LID in PD patients.

## Supplementary Information

ESM 1 (DOCX 32.9 kb MB)

## Data Availability

All data underlying the results are available as part of the article, and no additional source data are required.
