# Supplementary figures and images for: Mavoglurant (AFQ056) for the treatment of levodopa-induced dyskinesia in patients with Parkinson’s disease: a meta-analysis
Source: Neurol Sci. 2021 May 20;42(8):3135–43. doi: 10.1007/s10072-021-05319-7 (PMC8342336; doi:10.1007/s10072-021-05319-7)

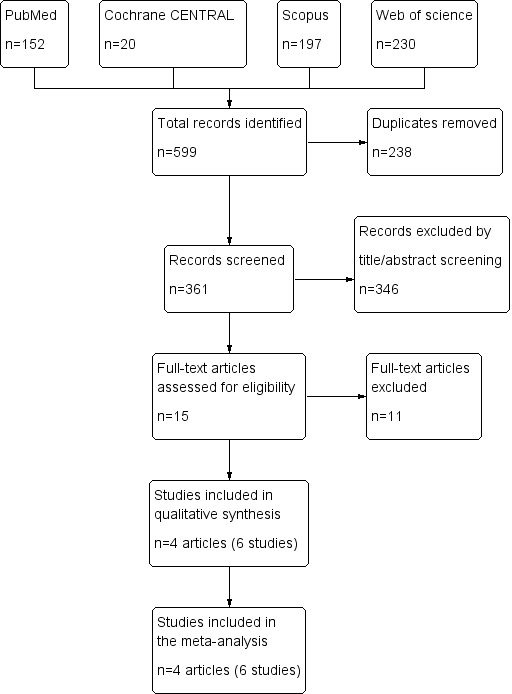


PRISMA flow diagram of the study selection process

Supplement: Supplementary file 1 — (DOCX 32.9 kb MB) [file 10072_2021_5319_MOESM1_ESM.docx]
